# Management of screwdriver-induced penetrating brain injury: a case report

**DOI:** 10.1186/s12893-016-0195-5

**Published:** 2017-01-10

**Authors:** Jia Shi, Yumin Mao, Jiachao Cao, Bo Dong

**Affiliations:** Department of Neurosurgery, The Third Affiliated Hospital of Soochow University, Changzhou City, 213003 China

**Keywords:** Screwdriver, Penetrating brain injury, Right zygomatic bone, Cranitomy

## Abstract

**Background:**

Penetrating brain injury (PBI) can be caused by several objects ranging from knives to chopsticks. However, an assault with long and electric screwdriver is a peculiar accident and is relatively rare. Because of its rarity, the treatments of such injury are complex and nonstandardized.

**Case presentation:**

We presented a case of a 54-year-old female who was stabbed with a screwdriver in her head and accompanied by loss of consciousness for 1 h. Computer tomography (CT) demonstrated that the screwdriver passed through the right zygomatic bone to posterior cranial fossa. Early foreign body removal and hematoma evacuation were performed and the patient had a good postoperative recovery.

**Conclusions:**

In this study, we discussed the clinical presentation and successful management of such a unique injury caused by a screwdriver. Our goal is to demonstrate certain general management principles which can improve patient outcomes.

## Background

Intracranial injuries caused by penetrating foreign bodies are associated with high risks of morbidity and mortality due to relevant infection, seizures, vascular injury and cerebrospinal fluid leakage [1, 2]. Penetrating brain injury (PBI) in civilian population can be caused by almost all sharp and blunt objects, ranging from knives and scissors to chopsticks and screwdrivers [1]. To date, PBI with a screwdriver is a peculiar accident and is relatively rare [3].

Screwdriver-induced PBI is severe and neglected [4, 5]. First, due to their length and spiral force, the tip of rigid screwdrivers may be enable to penetrate into the calvarium, and once through the bone, the screwdrivers may pivot around the entry point in the skull and then cause curved intracranial injuries, which are far more serious than the damage of skin surface. Second, if the screwdriver is withdrawn, the small entry wound may be missed by clinical examination, and the seriousness of intracranial injuries may also be overlooked [6]. Therefore, it is relatively complex to manage such injuries owing to their rarity and characteristics.

In this study, we discussed the clinical presentation and successful management of such an unique injury caused by screwdriver. Our goal is to demonstrate certain general management principles which can improve patient outcomes. It is worth mentioning that this case report strictly adhered to care guidelines.

## Case presentation

### History

**Fig. 1:**
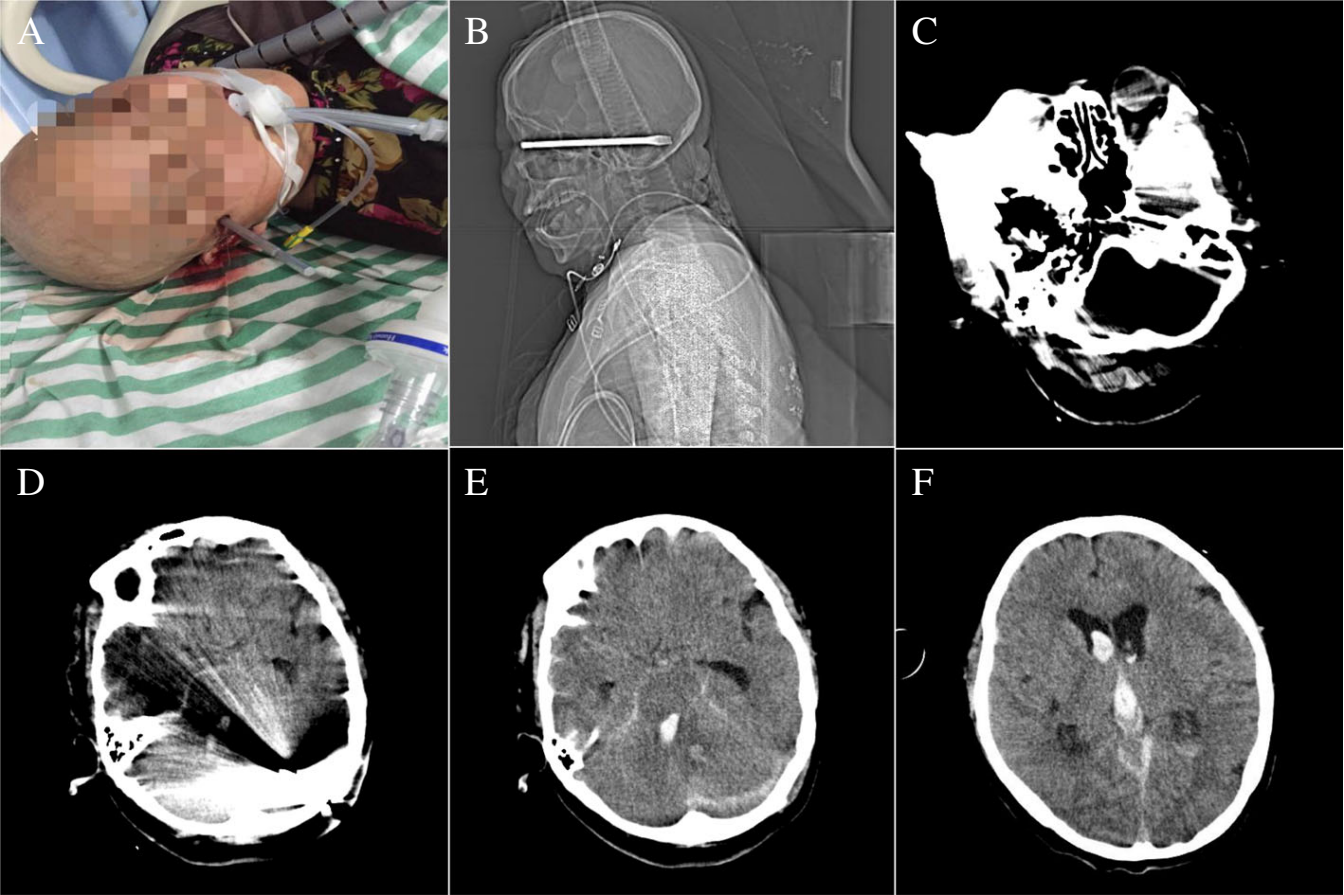
**a**. Clinical picture showing a strewdriver penetrating the right cheekbone. **b**. Computed tomogram (CT) of brain showing the metallic foreign body with metal artefacts. **c**-**d**. CT of head-brain window showing a metallic foreign body passed through the right cheekbone to posterior cranial fossa. **e**-**f**. CT scan showing left cerebellar hematoma and subarachnoid hemorrhage

A 54-year-old-female patient, without addictions or comorbidities, was admitted in our emergency department due to assault with a screwdriver in her head and loss of consciousness for 1 h. Her colleagues explained that she suffered the injury hit by shedding electric screwdriver when working in a factory, and the screwdriver penetrated the right zygomatic bone (Fig. 1a). The injury was associated with an immediate coma and bleeding of the right ear. The patient had no history of vomiting or seizures. Owing to the disorder of vital signs, the patient underwent tracheal intubation in emergency department.

### Examination

Neurological examination revealed a Glasgow Coma Scale (GCS) of 3/15. A screwdriver was seen partially penetrating the right cheekbone through a lacerated horizontal wound of 10 mm × 10 mm. The injury was accompanied by left pupillary dilatation (5 mm) with disappeared responsive to light, right pupillary dilatation (3 mm) and unresponsive to light, and bleeding of right ear. There was no evidence of any other injury.

A noncontrast axial computed tomographic (CT) scan showed a metallic foreign body passing through the right cheekbone to posterior cranial fossa, and a linear structure extending from right temporal bone, petrous bone, middle cranial fossa, posterior fossa to left occipital bone (Fig. 1b-c). Meanwhile, left cerebellar hematoma and subarachnoid hemorrhage were also demonstrated in CT scan (Fig. 1e-f). Because of the clinical status of the patient, a digital subtraction angiogram could not be performed.

### Operation

**Fig. 2:**
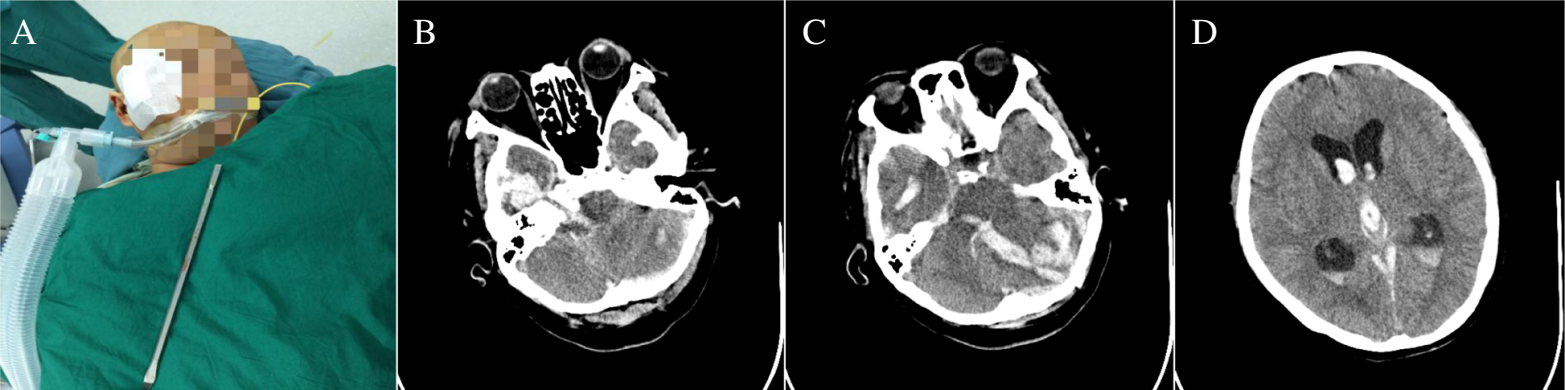
**a**. Clinical picture showing the strewdriver removed surgically. **b**. CT of head-brain window showing hematoma of strewdriver tract. **c**. CT scan showing a left cerebellar contusion with hematoma and obvious compression of the fourth ventricle and brain stem. **d**. CT scan showing high density corrosion cast of the ventricular system

The patient was emergently taken to the operating room (OR) and immediately given broad-spectrum antibiotic coverage with penicillin (3.75 g, ivgtt). The patient was given a right cheekbone craniectomy and removal of the foreign body at supine position. 6 cm long straight incision was adopted with the entry point as the center. Craniotomy was performed along the skin incision, during which the screwdriver came along and was removed (Fig. 2a). Afterwards the wound was debrided, cleaned, and closed. Brain CT scan was made again and revealed little hematoma of screwdriver tract, a left cerebellar contusion with about 15 ml hematoma, obvious compression of the fourth ventricle and brain stem, and intraventricular hemorrhage (Fig. 2b-d). Then right frontal puncture was immediately performed, followed by drainage of cerebrospinal fluid (CSF), which was as higher as 20 mmHg, to achieve maximum brain relaxation. The patient was then repositioned, placed in prone position and given evacuation of posterior fossa hematoma. The incision was made along the median line, about 16 cm, then skin and muscle flap were separated and the skull was exposed. A 6 cm × 5 cm flap was removed from occipital bone and about 15 ml of subdural hematoma was evacuated. Contusive cerebellar tissues and sutured dura were removed. The patient was then transferred to the neurosurgery intensive care unit (NICU) for postoperative management.

### Postoperative outcome

**Fig. 3:**
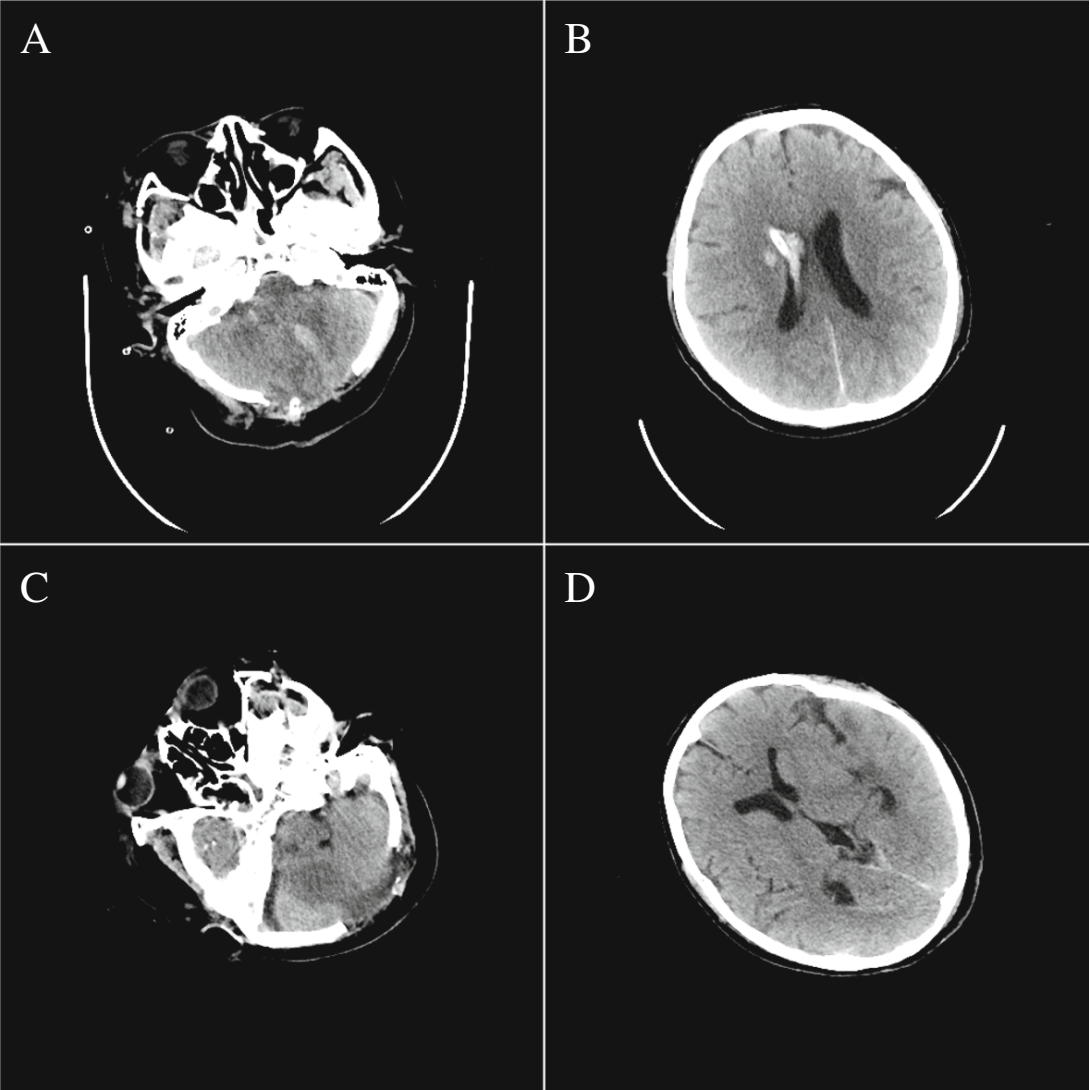
**a**-**b**. CT of brain taken on post-operative day 2, showing smaller posterior fossa hematoma and drainage of CSF. **c**-**d**. CT of brain taken on post-operative day 42, showing complete absorption of haematomas and normal ventricular system

On post-operative day (POD) 2, brain CT showed smaller posterior fossa hematoma. Meanwhile, external ventricular drainage tube was also placed to replace CSF and prevent obstructive hydrocephalus (Fig. 3a-b). However, the patient suffered persistent coma, with GCS of 4/15, complicated with pulmonary infection in the subsequent 3 days. Tracheotomy was performed to open the airway and control infection. Meanwhile, the patient received aggressive broad-spectrum antibiotics which were maintained for 14 days. Intravenous sodium valproate was given for prophylaxis against posttraumatic seizures. No other serious complications were reported after symptomatic supportive measures such as anti-infection treatments and nerve nutrition. The patient’s GCS score was improved to 12 on POD 42. Postoperative brain CT scan (Fig. 3c-d) revealed favorable findings. The patient was discharged on POD 60. Postoperative 6-month follow-up evaluation showed normal neurological status of the patient, without further seizures.

## Discussion

PBI involving foreign bodies is less prevalent than closed head injuries but often causes a worse prognosis. To date, there is no standardized management for such injuries because different injury patterns share with each scenario. However, some general management principles can be applied to almost any case to improve patient outcomes.

First is the preoperative examination. Head CT is the most sensitive imaging modality for PBI, to identify the extent of bone and parenchymal injuries and formulate operation plan [7]. In case of suspicion for vascular injury, CT cerebral angiography is also needed to evaluate traumatic aneurysm, which may rapidly develop after PBI [8].

Second is the operative management. In this study, the patient had severe low GCS of 3/15, which could be attributed to 2 points: 1) The characteristics of PBI caused by a screwdriver. 2) The patient presented with posterior fossa hematoma in the early stage, which might oppress brainstem, leading to disorder of vital signs. However, timely and effective surgical interventions made the patient have a good prognosis [9, 10]. Generally, the goals of surgical intervention for such injury are to: 1) Remove the penetrating object and accompanying necrotic debris around the injury site. According to our study, we recommended removal of the screwdriver through its trajectory with minimum injury. However, in some cases, foreign bodies were removed roughly on the spot, which would lead to bleeding of puncture and poor prognosis [6, 11]. 2) Eliminate any hematomas developed from the injury. 3) Ensure watertight closure of the dura and prevent CSF leakage [12].

Third is the postoperative management. 1) Prophylactic antibiotics and antiseizure medications are recommended to be applied for the first week [13, 14]. In this case, we prolonged the use of antibiotics owing to deep penetrating tract and pulmonary infection. 2) For severe PBI patient with postoperative coma and pulmonary infection, tracheotomy could be help to prevent the damage of chronic hypoxia on brain tissue and strengthen the management of respiratory tract. 3) Postoperative imaging and follow-up are important to evaluate complications such as pulmonary infection, delayed intracranial hematoma and posttraumatic hydrocephalus, which can be presented in a delayed mode [8].

However, there are still some limitations in this case report. First, the patient with a metallic foreign body in her head was unable to perform magnetic resonance imaging (MRI). Meanwhile, because of obvious CT imaging artifacts, computed tomography angiography (CTA) imaging and three-dimensional reconstruction could not be used to identify the relationship between metallic foreign body and intracranial vessels or skull. Second, the patient’s GCS score on arrival was 3/15 with unstable vital signs and formation of traumatic cerebral hernia. Thus immediate surgical decompression was needed, which kept us from digital subtraction angiography and made us unable to clear intracranial vascular injury. Third, the would tract induced by screwdriver was deep and long, involving multiple lobes,which made it difficult to thorough debridement. Therefore, it may result in residues of foreign bodies or necrotic tissue and increase the risk of infection.

## Conclusions

In conclusion, this is a unique case of penetrating screwdriver injury. Despite the removal of the screwdriver from the intracranial tissue through the orbit, we achieved positive outcomes in this challenging case due to the ability of the neuro trauma unit and the cooperation of multidisciplinary team, involving neurosurgeons, emergency physicians, radiologists and anesthesiologists.

## Abbreviations

CSF: Cerebrospinal fluid
CT: Computer tomography
GCS: Glasgow coma scale
NICU: Neurosurgery intensive care unit
PBI: Penetrating brain injury
POD: Post-operative day
